# Assessment of Surgical Quality in Radical Prostatectomy: Review of Objective Intraoperative and Functional Evaluation Scales

**DOI:** 10.3390/jcm14217458

**Published:** 2025-10-22

**Authors:** Jakub Kempisty, Krzysztof Balawender, Oskar Dąbrowski, Karol Burdziak

**Affiliations:** 1 Klinika Urologii i Urologii Onkologicznej, Uniwersytecki Szpital Kliniczny im. Fryderyka Szopena w Rzeszowie, ul. Szopena 2, 35-055 Rzeszow, Poland; 2 Department of Surgery, Faculty of Medicine, Medical College of Rzeszow University, al. mjr. W. Kopisto 2a 35-359 Rzeszow, Poland; 3 Department of Normal and Clinical Anatomy, Medical College of Rzeszow University, 35-615 Rzeszow, Poland

**Keywords:** radical prostatectomy, robotic surgery, surgical quality, intraoperative assessment, functional outcomes, nerve-sparing, surgical education

## Abstract

Radical prostatectomy remains a cornerstone treatment for localized prostate cancer. While oncological control is essential, functional outcomes such as urinary continence and erectile function play a critical role in patient satisfaction and quality of life. Despite the growing emphasis on surgical quality, no standardized intraoperative scoring system has been universally adopted. This narrative review summarizes current approaches to evaluating the technical quality of radical prostatectomy and associated functional outcomes. It focuses on objective intraoperative assessment tools and functional evaluation scales used in clinical research and surgical education. A non-systematic literature search was conducted using the PubMed and Scopus databases to identify relevant intraoperative assessment tools (e.g., GEARS, PACE, and OSATS), functional scales (e.g., IIEF, EPIC, and pad test), and outcome reporting systems. Articles were reviewed for scale structure, clinical applicability, validation status, and limitations. Several tools have been developed to evaluate surgical skills in minimally invasive surgery, yet few are specific to radical prostatectomy. Most rely on subjective surgeon assessment or delayed functional outcomes, limiting their utility for intraoperative feedback. Video-based assessment is promising but underutilized. A gap remains for a prostatectomy-specific, reproducible, and real-time assessment scale. There is a pressing need for validated tools that bridge the gap between surgical technique and functional outcomes. Current methods lack specificity and reproducibility. Development of an objective, intraoperative scoring system may support surgeon feedback, quality improvement, and improved patient counseling.

## 1. Introduction

Radical prostatectomy remains the gold standard treatment for localized and locally advanced prostate cancer, offering excellent oncological control in appropriately selected patients [1]. In many regions, it is the most frequently performed definitive therapy for prostate cancer, reflecting its established role in durable cancer control. Over time, however, the definition of success has evolved. Whereas oncological outcomes such as cancer-specific survival once dominated treatment evaluation, functional recovery, particularly urinary continence and erectile function, has become equally important [2,3].

Despite substantial improvements in surgical technology, especially the widespread adoption of robot-assisted radical prostatectomy (RARP), variability in functional outcomes persists even among high-volume centers and experienced surgeons [4,5]. This variability underscores the importance of technical factors. Subtle differences in the dissection of neurovascular bundles, vesicourethral anastomosis, and hemostasis can markedly influence postoperative recovery [6]. Although robotic platforms have improved visualization and ergonomics, they do not eliminate variability in human performance. This heterogeneity highlights the influence of surgical technique on functional outcomes.

Traditionally, surgical quality has been evaluated retrospectively using long-term outcomes such as recovery of potency, continence, or positive surgical margins. These measures are essential but delayed and multifactorial. Age, comorbidities, baseline urinary and sexual function, and tumor stage all influence outcomes, obscuring the contribution of intraoperative technique [7]. Although outcome-based evaluation remains foundational, it cannot provide timely, surgeon-specific feedback to improve technique on a day-to-day basis.

Consequently, there is growing interest in developing objective intraoperative assessment tools for real-time feedback, structured training, and standardized quality control [8,9,10]. Such instruments would benefit trainees by offering targeted, constructive feedback—and experienced surgeons by facilitating self-assessment, benchmarking, and continuous quality improvement.

Several systems have been proposed. Among the most widely recognized are GEARS (Global Evaluative Assessment of Robotic Skills) [11], OSATS (Objective Structured Assessment of Technical Skills) [12], and PACE (Prostatectomy Assessment and Competency Evaluation) [13]. GEARS provides a structured framework for robotic skills such as depth perception, dexterity, efficiency, and bimanual coordination. OSATS, initially developed for open and laparoscopic procedures, uses structured checklists and global rating scales. PACE represents an attempt to create a more procedure-specific tool tailored to radical prostatectomy. Yet each instrument faces limitations preventing routine adoption.

Generic tools such as GEARS and OSATS provide insight into general surgical competency but do not capture prostatectomy-specific nuances such as neurovascular bundle dissection, bladder neck preservation, or posterior rhabdosphincter reconstruction. More specialized tools like PACE offer procedure-specific guidance but are time-consuming, heavily dependent on retrospective video review, and insufficiently validated across institutions.

Video-based assessment shows particular promise, allowing detailed retrospective evaluation of intraoperative technique. Studies demonstrate that trained reviewers can identify subtle differences in tissue handling or dissection that may influence outcomes [14,15]. Video libraries also create opportunities for structured feedback, educational case discussions, and machine learning-based analytics. However, video review remains underutilized due to resource demands, lack of standardized frameworks, and concerns about feasibility in high-volume settings. Without a universally accepted scoring system, assessments remain fragmented and subjective.

Radical prostatectomy thus presents a paradox. The field leads in technological innovation with robotic platforms, advanced imaging, and digital tools, yet lacks a universally adopted method to evaluate surgical performance objectively and reproducibly. This gap hampers research correlating intraoperative technique with outcomes and limits opportunities for surgical education and quality assurance.

The challenge is magnified in training. For young urologists, radical prostatectomy demands mastery of oncological principles and delicate functional preservation. Training traditionally occurs through mentorship and case exposure, with feedback often subjective and inconsistent. Without structured intraoperative assessment tools, trainees may struggle to identify areas needing improvement or to benchmark their progress. A standardized scoring system could provide clarity, helping focus on critical steps and avoid common pitfalls early in the learning curve.

For experienced surgeons, structured assessment offers an avenue for continuous professional development. Increasing transparency, benchmarking, and outcome reporting drive quality improvement. Validated tools could enable objective documentation of performance, demonstrate quality, and facilitate peer comparison. In the absence of standardized intraoperative assessment, variability persists, and institutions lack a reliable method to monitor or ensure surgical quality beyond long-term outcomes.

From a systems perspective, objective assessment tools also support quality assurance, accreditation, and health policy. Prostate cancer remains one of the most common malignancies worldwide, and even small improvements in surgical quality can yield significant benefits, fewer complications, faster recovery of continence and potency, and reduced healthcare costs. Standardized surgical quality assessment is essential to ensure equitable access to high-quality surgery across regions and institutions.

Finally, the development of robust, prostatectomy-specific assessment tools aligns with advances in digital health and artificial intelligence. As surgical video capture becomes routine and machine learning algorithms become more sophisticated, opportunities emerge to integrate automated analysis into intraoperative assessment. Such tools could eventually provide real-time feedback, highlight deviations from optimal planes of dissection, or quantify technical metrics such as efficiency, precision, or nerve-sparing fidelity. Successful implementation, however, depends on establishing standardized frameworks against which machine-derived metrics can be trained and validated.

In summary, radical prostatectomy is at a pivotal point where oncological efficacy must be matched by functional preservation and demonstrable surgical quality. Current assessment methods remain fragmented, retrospective, and variably applicable to daily practice. There is an urgent need for objective, intraoperative, and procedure-specific scoring systems to provide timely feedback, enhance education, and harmonize surgical standards. This review summarizes the current state of objective surgical assessment tools in radical prostatectomy, focusing on intraoperative scoring methods, functional evaluation scales, and limitations in correlating technique with outcomes, and highlights the rationale for a dedicated, prostatectomy-specific quality assessment system.

## 2. Materials and Methods

Because the available literature on intraoperative and functional assessment tools in radical prostatectomy is heterogeneous in design, endpoints, and validation status, we opted for a non-systematic narrative review. This approach allows a focused and critical appraisal of the most relevant studies rather than a comprehensive systematic review.

We searched PubMed and Scopus for articles published between January 2000 and May 2025 using combinations of the terms “radical prostatectomy,” “robot-assisted radical prostatectomy,” “surgical quality assessment,” “intraoperative scoring,” “functional outcomes,” “GEARS,” “OSATS,” “PACE,” “IIEF,” and “EPIC”. Reference lists of key articles were hand-searched to identify additional studies. Articles were included if they addressed intraoperative or functional assessment tools relevant to radical prostatectomy, contained information on scale structure or validation, or reported clinical applicability. No PRISMA protocol was applied.

## 3. Types of Surgical Quality Assessment in Radical Prostatectomy

Radical prostatectomy is a complex procedure requiring meticulous technique, particularly during nerve-sparing dissection and reconstruction of the vesicourethral anastomosis. Small variations in surgical precision can translate into significant differences in both oncological and functional outcomes. Assessing the quality of this operation can therefore be approached from several perspectives: intraoperative evaluation, postoperative functional outcomes, and oncological or pathological results. Each reflects a distinct dimension of surgical performance and carries its own strengths and limitations in terms of reliability, timeliness, and relevance to both surgeons and patients.

## 4. Intraoperative Assessment Tools

Intraoperative assessment aims to capture the quality of the surgeon’s technique in real time or shortly after the procedure. Tools such as GEARS (Global Evaluative Assessment of Robotic Skills) and OSATS (Objective Structured Assessment of Technical Skills) were developed to provide structured evaluation of surgical skills across multiple specialties [16,17]. These frameworks typically focus on general domains such as efficiency, depth perception, dexterity, and tissue handling. Their structured nature allows for standardized scoring across trainees and surgeons, which is useful for educational purposes. However, while these instruments have value in assessing generic surgical competencies, their utility in daily clinical practice for radical prostatectomy is limited.

The main limitation is that GEARS and OSATS were not designed specifically for radical prostatectomy and therefore fail to capture the anatomical nuances unique to this procedure. For example, they do not account for the precision required during neurovascular bundle handling, the preservation of fascial planes, or the technical accuracy of dissection around the apex of the prostate and bladder neck. These steps are crucial in determining continence and erectile function recovery, yet they fall outside the scope of general skill assessments. Additionally, their application in a real-time intraoperative setting can be logistically challenging, as it requires the presence of trained observers and structured scoring at the console or bedside. This makes routine implementation impractical outside of research or training environments.

## 5. Procedure-Specific Instruments

Recognizing these limitations, researchers have attempted to develop more tailored tools that capture the technical nuances of radical prostatectomy. Among these, PACE (Prostatectomy Assessment and Competency Evaluation) represents one of the first procedure-specific efforts [13]. PACE aims to provide structured scoring that reflects key steps of radical prostatectomy, such as bladder neck dissection, nerve-sparing technique, and urethrovesical anastomosis. While procedure-specific tools such as PACE represent a clear advancement, their integration into daily surgical practice has been slow.

One of the primary reasons is that PACE and similar instruments are heavily dependent on video-based review. This approach, while valuable for capturing surgical detail, is resource-intensive and requires significant time investment from both reviewers and institutions. Furthermore, video analysis is inherently retrospective, meaning that feedback to the surgeon is delayed and cannot directly guide intraoperative decision-making. These limitations restrict widespread adoption and prevent procedure-specific tools from fulfilling their potential as real-time quality assurance mechanisms.

Another challenge is the lack of external validation and consensus on scoring thresholds. While a video review system may provide meaningful feedback within a single institution or training program, its reproducibility across centers and surgical teams remains uncertain. Without standardized benchmarks, comparisons between surgeons or institutions are difficult, limiting the broader applicability of these tools in quality assurance programs.

## 6. Functional Outcome Measures

Functional assessment tools remain an essential component of evaluating the quality of radical prostatectomy, as they reflect the long-term impact of surgical technique on patient well-being. Instruments such as the International Index of Erectile Function (IIEF), pad count for urinary continence, and the Expanded Prostate Cancer Index Composite (EPIC) are widely used in both clinical practice and research to capture patient-reported outcomes [18,19,20]. These measures are indispensable for assessing recovery of sexual and urinary function, which represent the most relevant quality-of-life domains after surgery.

However, while functional outcomes are vital, they are inherently retrospective and highly patient dependent. Recovery of continence and potency can be influenced by baseline function, comorbidities, age, preoperative counseling, and psychosocial factors. As a result, functional scores provide only indirect information about the quality of intraoperative technique. Moreover, the feedback they provide to the surgeon comes months or even years after the operation, which limits their utility for immediate improvement or real-time skill refinement.

## 7. Oncological and Pathological Endpoints

In addition to intraoperative and functional assessments, oncological outcomes such as margin status, extracapsular extension, and lymph node yield have traditionally been used as markers of surgical quality. Positive surgical margins, for instance, are frequently cited as a surrogate endpoint for oncological adequacy [21]. However, while these measures provide important information about cancer control, they too are limited by multifactorial influences. Tumor biology, preoperative risk stratification, and patient selection play a significant role in determining oncological outcomes, which means that margin status cannot serve as a pure measure of technical skill. Similarly, pathological endpoints cannot reliably reflect the quality of nerve-sparing or reconstructive steps, which are essential for functional preservation [22].

## 8. The Unmet Need for Standardization

Taken together, the heterogeneity of available frameworks reflects a broader issue: there is currently no universally accepted, validated, and reproducible system for intraoperative evaluation of nerve-sparing quality in radical prostatectomy. In the absence of such a tool, surgical education, benchmarking, and institutional quality assurance remain fragmented. Trainees are often guided by subjective feedback from mentors, while experienced surgeons rely on personal impressions or delayed functional results to gauge their performance. This lack of standardization creates inconsistencies in surgical training and perpetuates variability in outcomes.

The development of a universally accepted intraoperative scoring system would provide multiple benefits. It would allow for structured and reproducible feedback during training, help avoid the transmission of poor technical habits, and facilitate cross-institutional comparisons. Moreover, it would create a framework for integrating novel technologies such as artificial intelligence, which could eventually automate parts of intraoperative assessment. Until such a system is developed and validated, the evaluation of surgical quality in radical prostatectomy will remain fragmented, and opportunities to improve both training and patient outcomes will continue to be missed (Table 1).

## 9. Discussion

Training and learning curves play a pivotal role in the quality of radical prostatectomy. Recent studies highlight how structured assessment can shorten the time to proficiency and improve functional outcomes. Predictors of trainees’ proficiency during the learning curve of robot-assisted radical prostatectomy at high-volume institutions, further demonstrated that objective evaluation of performance and standardized feedback can accelerate skill acquisition and reduce variability among surgeons. Cumulative sum (CUSUM) analyses have also been successfully applied to robot-assisted radical prostatectomy to monitor performance over time. Multicenter video review panels and benchmarking initiatives have emerged, providing richer datasets and reducing institutional bias. In parallel, machine learning techniques are expanding beyond simple step recognition to include metrics such as tissue handling, energy use, and dissection precision. Together, these developments underscore the need for standardized intraoperative scoring systems that can be applied across centers and linked to objective functional outcomes.

Despite the promise of available tools, significant challenges remain. Procedure-specific instruments such as PACE have not gained widespread traction because of their resource-intensive nature, reliance on retrospective video review, and limited external validation. Inter-rater variability further complicates video-based scoring, with differences in reviewer experience leading to inconsistent assessments. Moreover, the predictive validity of GEARS scores for functional outcomes has not been conclusively established. Major clinical guidelines—including those of the EAU, AUA, and NCCN—currently offer little or no formal guidance on intraoperative quality assessment in radical prostatectomy [23,24]. This lack of standardized recommendations highlights the urgent need for a validated, reproducible, and clinically meaningful scoring framework.

Functional outcome measures, including the IIEF-5, EPIC, and pad count, remain essential benchmarks for postoperative quality of life. However, these tools are inherently retrospective. They provide feedback months after surgery, are heavily influenced by patient-specific factors such as age and baseline function, and offer no intraoperative guidance for quality improvement. Consequently, they are invaluable for assessing patient outcomes but insufficient for guiding surgical performance in real time.

This fragmented landscape underlines the critical unmet need for a validated, reproducible, and procedure-specific intraoperative scoring system for radical prostatectomy. An ideal scale would combine anatomical specificity, reproducibility across raters and institutions, feasibility for intraoperative or immediate postoperative use, and correlation with both short- and long-term outcomes. Existing systems fall short on one or more of these criteria, preventing their universal adoption.

Standardized scoring systems would hold enormous value for education, allowing trainees to receive explicit, objective feedback and helping experienced surgeons engage in continuous quality improvement. At the institutional level, intraoperative scoring would facilitate benchmarking and quality assurance. For patients, it would enhance counseling and set realistic expectations. Emerging technologies such as AI-driven video analysis and virtual reality simulation may accelerate the development of such systems by automating evaluation and reducing dependence on human raters.

## 10. Future Directions

The future of surgical quality assessment in radical prostatectomy will likely be shaped by the integration of artificial intelligence (AI), video-based evaluation, and advanced simulation platforms. AI algorithms have already demonstrated the ability to autonomously interpret surgical video, recognizing procedural steps and identifying the use of surgical instruments with high accuracy [25]. Specific applications in robot-assisted radical prostatectomy include phase recognition and intraoperative event labeling, with recent systems achieving over 90% accuracy in detecting surgical steps such as dissection and anastomosis [26,27]. These tools may soon provide real-time intraoperative feedback, bridging the gap between technical performance and functional outcomes.

Simulation-based education is also evolving rapidly. Virtual reality (VR) simulators and augmented training platforms allow surgeons to rehearse RARP procedures in a safe and controlled environment. Several studies have confirmed their educational value, demonstrating significant improvements in robotic console performance and skill acquisition after structured VR training programs [28,29]. Comparative studies suggest that VR simulators are at least as effective as physical bench-top models, while offering additional opportunities for objective assessment and scalable distribution [30].

Beyond education, integration of AI and VR into training and clinical workflows may support standardization and proficiency-based certification. By combining intraoperative video analysis with validated scoring systems, surgical education can shift from subjective mentoring toward objective, data-driven evaluation. This approach may reduce variability across institutions, enhance patient safety, and accelerate the learning curve for younger surgeons [31].

Ultimately, the convergence of AI, big data, and immersive simulation represents a promising pathway toward a reproducible, transparent, and outcome-oriented system for surgical quality assessment in radical prostatectomy.

## 11. Conclusions

Radical prostatectomy remains one of the most technically demanding procedures in urologic oncology, where functional outcomes such as continence and erectile function depend strongly on surgical technique. Despite major advances in robotic technology and training, no universally accepted intraoperative scoring system currently exists. Existing tools such as GEARS and OSATS are valuable in training and simulation but are too generic to capture prostatectomy-specific nuances, while PACE and similar procedure-specific instruments remain resource-intensive and limited mainly to video review. Functional outcome scales, although indispensable for patient-centred evaluation, are retrospective and provide no real-time feedback to guide intraoperative performance.

Developing a validated, reproducible, and prostatectomy-specific intraoperative assessment scale therefore represents both a challenge and an opportunity. Such a tool could provide immediate, objective feedback, clarify critical steps for trainees, and support continuous professional development for experienced surgeons. It would also facilitate benchmarking across institutions, enhance quality assurance, and improve patient counselling by linking intraoperative performance to functional outcomes.

Looking ahead, the integration of artificial intelligence, video-based analytics, and virtual reality simulation offers a promising pathway to standardised, data-driven evaluation of surgical quality. These technologies may enable real-time skill assessment, automated feedback, and scalable training environments that complement traditional mentorship.

A future prostatectomy-specific intraoperative scoring system should integrate several domains: fidelity of nerve sparing, precision of anatomical plane dissection, hemostasis and blood loss control, preservation of key structures (bladder neck and urethral length), and overall efficiency of the procedure. Validation should occur in multicenter studies with standardized training and inter-rater reliability testing. Linking these intraoperative metrics to functional outcomes would provide the most meaningful benchmark for quality and support the development of AI-driven automated assessment.

In summary, objective intraoperative quality assessment is no longer an academic aspiration but a clinical necessity. Establishing a simple, reproducible, and anatomically grounded scoring system would harmonise standards, improve education, and ultimately elevate patient outcomes in radical prostatectomy worldwide.

## Figures and Tables

**Table 1:** Comparative Overview of Surgical and Functional Assessment Tools in Radical Prostatectomy.

Scale	Type	Scope of Use	Validation	Real-Time Use	Limitations	Relation to Functional Outcomes
GEARS	Intraoperative (technical)	Robotic surgery (all specialties)	Yes (video/simulation)	Limited	Not specific to prostatectomy	Indirect; limited evidence on prediction
OSATS	Intraoperative (technical)	General surgery, training	Yes (educational)	No	Too general	Indirect; generic skill domains
PACE	Intraoperative (RARP-specific)	Robotic prostatectomy	Partial	Limited (video-based)	Requires trained raters; lacks broad validation	Designed to correlate with functional steps but no large-scale proof
IIEF-5	Postoperative (functional)	Erectile function	Yes (clinical)	No	Subjective, delayed feedback	Direct measure of sexual function
EPIC	Postoperative (functional)	Urinary and sexual function	Yes (clinical)	No	Long questionnaire; requires compliance	Direct measure of QoL domains
Pad count	Postoperative (functional)	Urinary continence	Yes (practical)	No	Unstandardized threshold	Direct but delayed
Video-based scoring	Intraoperative (technical)	Prostatectomy technique (RARP)	No	Theoretically possible	No consensus; lacks validation	Potential but unproven link

## Data Availability

Not applicable.
